# The Use of Botulinum Toxin in a Case of Acquired Periodic Alternating Nystagmus

**DOI:** 10.22599/bioj.170

**Published:** 2021-04-15

**Authors:** Nadia Venturi, Gillian Adams, Maria Theodorou

**Affiliations:** 1 Moorfields Eye Hospital, GB

**Keywords:** abnormal head posture (AHP), nystagmus, visual acuity, oscillopsia

## Abstract

**Aim:**

To describe a case of acquired Periodic Alternating Nystagmus (PAN) with oscillopsia treated with botulinum toxin injections into four horizontal rectus muscles.

**Method:**

A 22-year-old woman presented with sudden onset PAN. The only abnormality found following extensive investigations was bilateral superior cerebellar peduncle atrophy on MRI. Various treatment options were discussed, with reasonable response to baclofen, less so to gabapentin. However, she was keen for a longer-term solution without medication-related adverse effects. She was offered weakening of all four horizontal rectus muscles recessions, either with botulinum toxin or surgery, and she opted for the former to simulate the effects of surgery. 2.5 units of Dysport were injected into each horizontal rectus muscle without adverse effect.

**Results:**

Off all treatment, Snellen Visual Acuity (VA) was 6/12 in either eye with oscillopsia as a result of the PAN. Post-botulinum toxin VA was 6/5 and 6/6 on the right and left respectively, with both subjective and objective improvement in the nystagmus and oscillopsia.

**Conclusion:**

Botulinum toxin has an important role in the nystagmus and strabismus clinics. Depending on the circumstances, it may be used as either long term treatment, or for surgical planning to simulate the effects of surgery. In this case, the effects were equivalent to high dose of baclofen and four horizontal rectus muscles recessions, which she underwent when the effects of the botulinum toxin had worn off. Botulinum toxin could be considered as a treatment option in acquired PAN, particularly in women of childbearing age and/or if intolerant or refractory to medical treatment, but ideally not as a long-term treatment option.

## Introduction

Periodic Alternating Nystagmus (PAN) is a horizontal jerk nystagmus that predictably oscillates in direction, amplitude, and frequency, with cycles in the acquired subtype typically lasting between two and four minutes. This is generally associated with a periodic alternating head turn to minimize the nystagmus, according to Alexander’s law. PAN may be congenital or acquired. The congenital form is often, but not exclusively, associated with albinism, while the acquired form is frequently associated with caudal brainstem or cerebellar disorders and/or medication, such as Lithium toxicity.

Acquired PAN is one of few subtypes of acquired nystagmus amenable to medical treatment, with baclofen having the most evidence for treatment (Comer, Dawson & Lee 2006; Halmagyi et al. 1980). Other medications have also been reported to be effective, including gabapentin (Shery et al. 2006), memantine (Kumar et al. 2009), clonazepam, 3,4-diaminopyridine, phenothiazine (Nathanson, Bergman & Bender 1953) and barbiturates.

The role of surgery in the treatment of acquired nystagmus is not well established although individual patients may benefit from large recessions of all horizontal rectus muscles due to the beneficial effects of mechanical weakening (Lee 2002; Miller, Subramanian & Patel 2016). The use of botulinum toxin in patients with acquired nystagmus has been described (Helveston & Pogrebniak 1988; Leigh et al. 1992; Ruben et al. 1994; Repka, Savino & Reinecke 1994; Tomsak et al. 1995; Ruben, Dunlop & Elston 1994; Thomas et al. 1996), mostly in the form of retrobulbar toxin, and less commonly in the horizontal muscles. In our practice (unpublished) we have also successfully used botulinum toxin for head postures, commonly recurrent head postures where horizontal rectus muscles have already been maximally recessed. It is difficult to form any definite conclusions from the literature because of the limited numbers of patients that have been reported, with different injection sites and variability in responses (Hobson and Rowe 2009).

To our knowledge, this is the first published case report successfully using botulinum toxin injections into all four horizontal rectus muscles in acquired PAN.

## Case Report

A 22-year-old female woke up in August 2015 with sudden onset of oscillopsia and jerky multi-directional head movement. She had no significant ocular history and no systemic signs on history or examination were detected. She had marked abnormal head movements, precluding accurate oculomotor assessment but a likely diagnosis of PAN was suspected clinically.

Previous bloods tests, including autoimmune, vasculitis, inflammatory, paraneoplastic, neoplastic and infective screen, were normal (except a low B12), as was lumbar puncture. Her stable Magnetic Resonance Imaging showed bilateral superior cerebellar peduncle atrophy, presumed to be the underlying aetiology.

Whilst being investigated, botulinum toxin was injected into the sternocleidomastoid muscles and the multi-directional head movements were abolished immediately and permanently. However, she was left with a marked alternating horizontal head posture which she utilised to dampen the PAN, which was now confirmed clinically.

Baclofen up to 90 mg/day was prescribed with improvement in the visual acuity and oscillopsia. Despite the large dose, this was tolerated without adverse effect. A trial of gabapentin was also initiated, but even at a dose of 600 mg/day the adverse effects of drowsiness was problematic. Memantine was also trialled, but without subjective, or minimal objective, improvement.

## Ophthalmic Findings

On baclofen 90 mg/day her Snellen VA with myopic spectacles and alternating head posture was 6/4 on the right and 6/5 on the left. She had a horizontal PAN with alternating null point with marked alternating head turn, adopted to dampen her nystagmus and oscillopsia. She had a well-controlled exophoria for near and distance with full motility. The rest of the examination was otherwise unremarkable. Off all medication, Snellen VA with glasses and AHP was 6/12 in each eye, with worsening of the nystagmus and oscillopsia.

Due to the increased risk of birth defects reported with the use of baclofen in early pregnancy (‘no authors listed’, 2015), and the preference to not be medication dependent, alternative treatment options were offered.

Alternative treatments discussed were: botulinum toxin injection to all four horizontal rectus muscles to simulate the effects of surgical intervention, although not previously reported in PAN in this form (Leigh et al. 1992); or maximal surgical recession of all horizontal rectus muscles, already reported effective in cases of PAN (Mimura et al. 2014; Hobson & Rowe 2009).

An informed decision was taken and, after discontinuation of all medications, a trial of botulinum toxin (2.5 units of Dysport Ipsen Ltd) was injected into to all four horizontal rectus muscles. Snellen visual acuity post injection was 6/5 on the right and 6/6 on the left eye, with a mild right ptosis and a subjective improvement in the oscillopsia and AHP with no restriction in motility.

**Figure 1:**
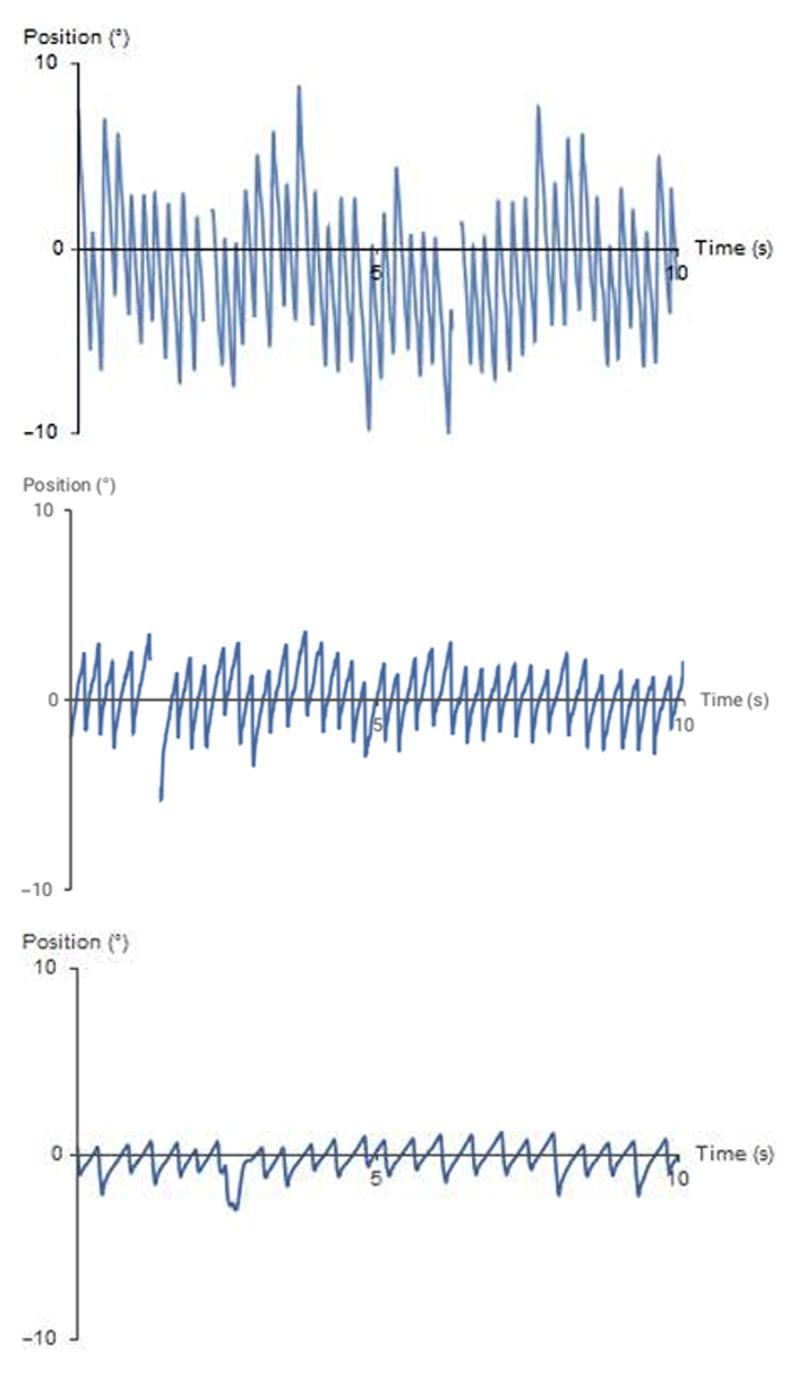
Position profile off all treatment, on baclofen and post-botulinum toxin into all four horizontal rectus muscles. A 10 second position profile off all treatment (upper figure), on baclofen (middle figure) and post botulinum toxin injection into four horizontal rectus muscles (lower figure), showing a marked reduction in both amplitude and frequency, resulting in an improvement in the slow phase of the nystagmus waveform.

Objective improvement was also confirmed with eye movement recordings, shown in ***Figure 1*** below. This shows a 10 second calibrated position profile for simplification.

The upper position profile, shown on the top, is pre-botulinum toxin and off all medication (i.e., baseline without treatment). It shows a high frequency high amplitude waveform with a linear slow phase with short foveation time (i.e., little time when the image is moving slowly over the fovea). Following both baclofen (middle) and botulinum toxin (lower) the frequency and amplitude is reduced, with an associated improvement in the foveation time more marked following botulinum toxin. Both treatments are associated with an improvement in visual acuity and oscillopsia.

Once the effects of botulinum toxin had worn off, the favourable effects gained from temporarily weakening the four horizontal rectus muscles resulted in recessing all four horizontal recti (medial recti -7mm, lateral recti -10mm). This allowed discontinuation of the medications. The effects of surgery are still maintained three years post operatively.

## Discussion

Standard treatment of PAN is typically pharmacological or surgical. Baclofen is commonly first line and tends to be more effective in acquired (rather than congenital) PAN. Baclofen is believed to work by activation of GABA receptors, specifically the GABA_B_ receptors (Halmagyi et al. 1980). Gabapentin may act on nystagmus as a GABA agonist (i.e. mimics the actions of GABA but does not bind to GABA receptors), a glutamate antagonist by inhibiting NMDA receptors, or via the voltage sensitive calcium channels, while memantine acts as a glutamate antagonist by inhibiting N-methyl d-aspartate (NMDA) receptors (Shery et al. 2006), although neither had significant effect on PAN in our case. The assumption is that PAN is more responsive to baclofen due to the effects on the GABA_B_ receptors. PAN is thought to arise from an abnormality of ‘velocity storage’ which is prolonged in PAN due to loss of inhibitory inputs from the cerebellar nodulus and uvula. It is thought that the inhibitory effects of GABAB receptors compensate for the loss of inhibitory inputs (Leigh & Zee 2015; Halmagyi et al. 1980).

Surgical intervention in the form of maximal symmetrical recessions of all four horizontal rectus muscles has been reported previously with success due to the mechanical effects on the nystagmus. This procedure is designed to symmetrically weaken the horizontal rectus muscles and mechanically reduce the amplitude of the nystagmus (Bietti and Bagolini 1960; Von Noorden & Sprunger 1991; Mimura et al. 2014; Hobson & Rowe 2009).

However, the use of botulinum toxin is relatively new in nystagmus (as opposed to strabismus). Botulinum toxin is a neurotoxin protein produced by the bacterium *Clostridium botulinum*. Injection of highly diluted doses of botulinum toxin into affected muscles temporarily prevents the release of acetylcholine from synaptic nerve terminals, blocking neuromuscular transmission, resulting in reduction in muscle activity without significant functional weakness (Nigam & Nigam 2010). The length of time for which the paralysis lasts depends on the individual, but it usually lasts for weeks, and occasionally months, before it wears off.

In nystagmus botulinum toxin is typically used for the management of strabismus (pre-op/long term) and to assess the risks of post-operative diplopia where the patient is thought to be high risk; it is a valuable adjunct to surgical treatment (Lennerstrand et al. 1998). Botulinum treatment may also be used to manage a horizontal or a chin up head posture (pre-op/long term) (Hobson & Rowe 2009), or as a retrobulbar injection in acquired nystagmus (Repka, Savino & Reinecke 1994; Ruben et al. 1994; Helveston & Pogrebniak 1988; Tomsak et al. 1995). Botulinum retrobulbar toxin injection is also used in PAN (Thomas et al. 1996). However, consideration must be given to the repeated risks of retrobulbar injections to one eye (with the resulting requirement for occlusion of the other eye) or both eyes.

In our practice we also offer toxin to adults who have previously had maximal surgery and recurrent head postures (horizontal or chin up). However, to our knowledge this is the first time it has been used in all four horizontal recti in PAN to successfully reduce the nystagmus and simulate the effects of surgical intervention. Most adverse effects are mild and reversible (Scott 1980). In this case the procedure was tolerated well with no significant effects.

## Conclusion

Injection of botulinum toxin directly into the extraocular muscles has been used in this case to treat an acquired PAN with subjective and objective improvement in visual acuity and oscillopsia; eye movement recording has shown improved nystagmus amplitude and frequency with a remarkable improvement in the slow phase. All these results have allowed the patient to remain off pharmacological treatment post operatively.

We have shown that botulinum toxin injection can play a role in the management of acquired PAN, but preferably to simulate the results of surgical treatment rather than as long-term treatment. Botulinum toxin has also proven to be a valuable alternative to baclofen treatment for our patient, being a woman of childbearing age.
